# Hydronephrosis and its causes identified on correlative fluoroscopy in a paediatric population at a resource-constrained tertiary hospital in Southern Africa

**DOI:** 10.4102/jcmsa.v4i1.351

**Published:** 2026-09-30

**Authors:** Faatimah Khan, Tanusha Sewchuran

**Affiliations:** 1 Discipline of Radiology, University of KwaZulu-Natal, Pietermaritzburg, South Africa

**Keywords:** hydronephrosis, paediatric urology, voiding cystourethrogram, posterior urethral valves, vesicoureteric reflux

## Abstract

**Background:**

Paediatric hydronephrosis has a high global prevalence. Identifying aetiologies is critical for planning investigations, management and preventing chronic renal impairment. This study reviewed the underlying causes of unilateral and bilateral hydronephrosis, specifically on voiding cystourethrogram (VCUG) examinations.

**Methods:**

A retrospective descriptive study (Jan 2021–June 2024) included children < 13 years who underwent VCUG following a sonographic diagnosis of hydronephrosis. Ultrasound and VCUG studies were retrieved from the institutional Radiology Information System. Data collected included demographics, hydronephrosis laterality and severity, VCUG and mercaptoacetyltriglycine-3 (MAG-3) results. Hydronephrosis was graded according to a dual grading system combining Radiology grading system and Onen’s criteria. Statistical significance was set at *p* < 0.05.

**Results:**

Of sixty-five patients included, 67.7% were male. Bilateral hydronephrosis occurred more frequently in males, while unilateral disease was more common in females. Grade III hydronephrosis was most prevalent. A cause was identified in 85.7% of bilateral vs. 43.2% of unilateral cases (*p* < 0.001). Posterior urethral valves (PUV) were the leading aetiology (36% bilateral; 14% unilateral; *p* = 0.035) followed by vesicoureteric reflux and bladder diverticula. Of 22 MAG-3 studies, 59% showed delayed excretion or reduced cortical function consistent with obstructive VCUG findings.

**Conclusion:**

Bilateral hydronephrosis correlated with higher severity grades, mostly due to posterior urethral valves. Severity grading highlights the importance of early detection and intervention to prevent renal failure. Resource constraints challenge SASPI treatment algorithm implementation, necessitating institution-specific imaging pathways.

**Contribution:**

This study highlights PUV as the primary aetiology of severe bilateral hydronephrosis and advocates for institution-specific, multidisciplinary imaging pathways.

## Introduction

Hydronephrosis is the dilatation of the urinary collecting system. Globally, there is a high prevalence of hydronephrosis in the paediatric population. Govender et al.,^1^ reported that the most frequent renal abnormalities detected were hydronephrosis and hydroureter, in a Johannesburg, South African setting. The prevalence of hydronephrosis ranges between 1%–5% in the foetus to 0.5% in the newborn.^2^

Obstruction of the pelvicalyceal system, i.e. the renal collecting system, results in various imaging manifestations and appearances, most importantly, hydronephrosis, which is the primary response to ureteropelvic junction (UPJ) obstruction and is termed ureteropelvic junction hydronephrosis (UPJHN). Persistent and severe obstruction with longstanding hydronephrosis may result in renal damage. In infants, the renal pelvis and calyces are adaptable to collecting system pressure changes. Compliance increases particularly when an extra renal pelvis is present, which is a normal variant anatomy. The calyces, however, are less compliant than the renal pelvis, with their dilatation implying a higher likelihood of hydronephrosis.^3^

The renal parenchyma comprises the cortex and medulla, as demonstrated in Figure 1. The latter has higher elasticity and quick compressibility compared to the renal cortex. In prolonged UPJ obstruction, its height decreases, resulting in loss of its normal morphology.^4^ The cortex is a vital component of renal function and is comparatively hard. Thinning of the cortex indicates renal injury as it is not affected by external factors (e.g. bladder filling, respiration, hydration or position), when compared to the pelvicalyceal system. It is therefore an objective imaging parameter to quantify renal damage. The recommended guideline for measurement is in the longitudinal plane at the thinnest renal parenchymal point, as it is less susceptible to intraobserver or interobserver variation.^3,4,5,6,7,8^

**FIGURE 1:**
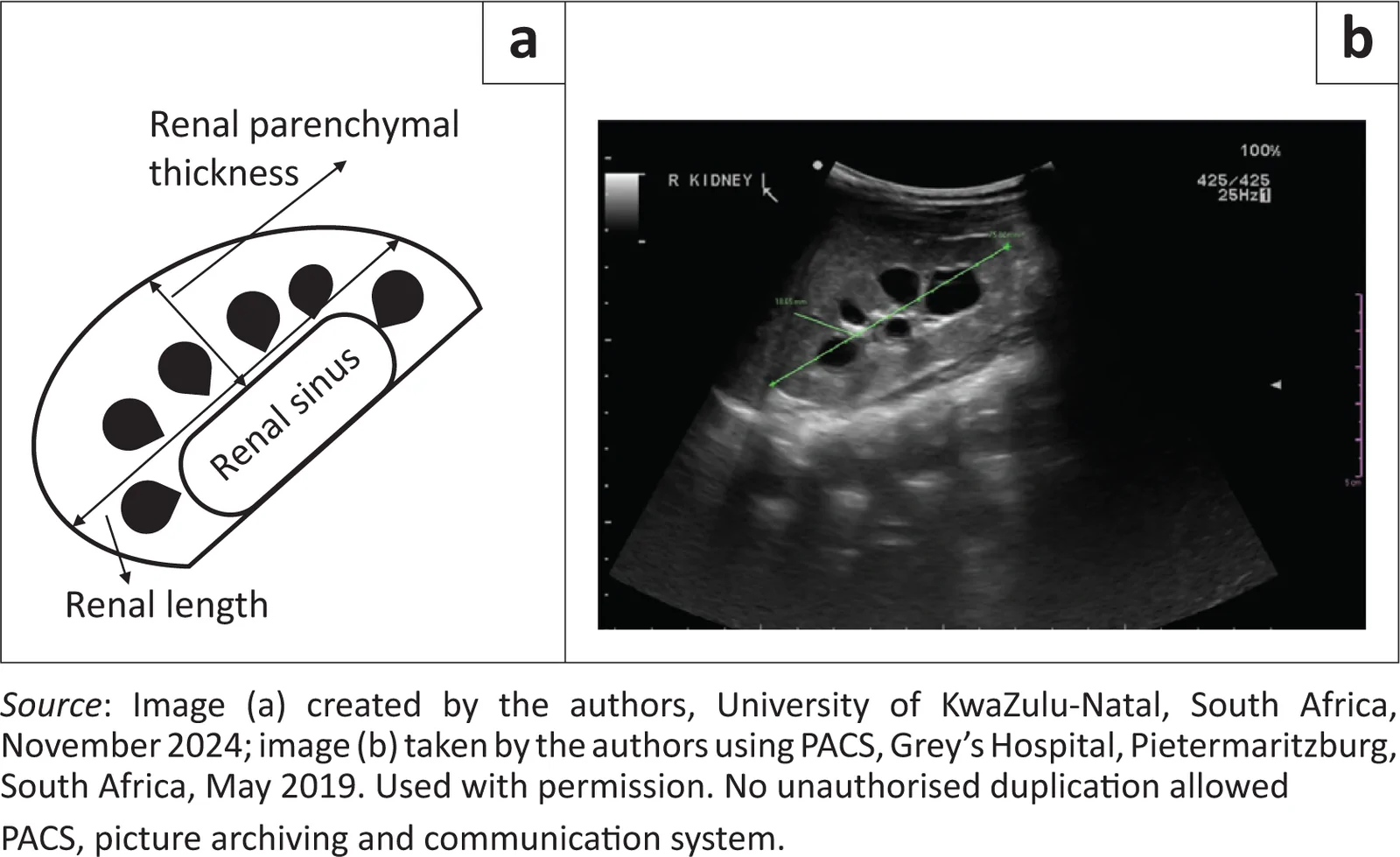
Unilateral hydronephrosis of the right kidney depicting renal length and renal parenchymal thickness in (a) schematic diagram and (b) sonographic image.

Not all cases of hydronephrosis are secondary to an obstructive cause. It is therefore important to re-assess newborns and neonates with prenatally diagnosed hydronephrosis, as many of these cases resolve.^3^ However, due to the decrease in glomerular filtration rate and other physiological changes in the newborn, it is imperative that follow-up ultrasound is performed at 4–6 months to ensure no recurrence or late features of obstruction have occurred. In cases of moderate or severe hydronephrosis detected on ultrasound or the presence of dilated ureter/s, a voiding cystourethrogram (VCUG) is recommended for evaluation of obstructive or non-obstructive aetiologies.^9^

According to studies in Johannesburg by Peterson et al.^10^ and in Finland by Visuri et al.,^11^ the most common cause of hydronephrosis is vesicoureteric reflux (VUR). In contrast, a study performed in the Johannesburg setting by Okoronkwo et al.,^12^ demonstrated posterior urethral valves (PUV) being the most common aetiology for hydronephrosis in the renal paediatric setting. In view of this, differences in the aetiology of hydronephrosis may exist at facility level. Knowledge of this is imperative for early detection of hydronephrosis and to assess the degree of chronicity. This will allow for prompt investigation, management and adequate follow-up. In view of the known ionising radiation exposure in fluoroscopic studies, it is further important to identify if follow-up is appropriate with ultrasound instead of repeated radiation exposure with unnecessary VCUG studies.

The aim of this study was to evaluate hydronephrosis in the paediatric population in a resource-limited setting, highlighting its causes, distribution and severity, using a combined radiology grading system.

## Research methods and design

### Study design and participants

This retrospective descriptive analysis was conducted at a tertiary referral centre between January 2021 and June 2024. The study included paediatric patients under 13 years who underwent voiding cystourethrograms following the diagnosis of unilateral or bilateral hydronephrosis. Patient data were collected from radiology request forms, reports and clinical records when available.

### Methodology

Only VCUG studies preceded by sonographic evidence of hydronephrosis were included in this study. Clinician request forms and radiology reports were obtained from the Radiology Information System (RIS), and corresponding images from both ultrasound and VCUG examinations were retrieved from the Picture Archiving and Communication System (PACS). All data were password-protected and only accessible to the authors of this study. Exclusion criteria included cases with no images on PACS or illegible request forms.

Sonographic images and VCUGs, along with their respective reports, were captured into an Excel spreadsheet for analysis. The spreadsheet included the patient’s age, gender, a brief history as per the documentation provided, sonographic parameters of renal parenchymal thickness and AP pelvis diameter for diagnosing hydronephrosis, unilateral or bilateral renal involvement, normal VCUG study results, details of abnormal results, as well as mercaptoacetyltriglycine-3 (MAG-3) diuretic renal scan and follow-up, availability permitting. The MAG-3 diuretic scan provided information of obstructive versus non-obstructive hydronephrosis, renal drainage dynamics and an evaluation of split renal function.

In addition, the imaging cascade for patients with recurrent urinary tract infections (UTIs) was assessed according to the South African Society of Paediatric Imaging (SASPI) guidelines.

### Statistical analysis

R version 3.6.3 of the R Core Team, 2020, a statistical computing and programming software, was used to produce descriptive and inferential statistics. Numerical variables were presented as five number summaries, including standard deviation and the coefficient of variation, depending on the distribution. Counts and percentage frequencies were used for summarising categorical variables with simple bar and multiple bar charts, and Likert plots were used for graphical displays. The numeric variables were further grouped with a variable with two categories, for which the mean differences were assessed using either *a t*-test or Wilcoxon, respectively, for the median, depending on the distributional properties. The applicable tests for categorical variables were either a Chi-square test or Fisher’s exact test, depending on the cell count. Post hoc tests for the categorical variables used row-wise paired *z*-tests. Binary logistic regression was used to assess factors associated with the outcome. A 5% level of significance was used as the benchmark to determine statistically significant differences or associations.

### Ethical considerations

Ethical clearance to conduct this study was obtained from the University of KwaZulu-Natal Biomedical Research Ethics Committee (No: BREC/00007666/2024), and site approval was obtained from Grey’s Hospital. Informed consent was not required for this study.

## Results

Three patients were excluded from the study due to no or inadequate imaging data.

A final total of 65 patients were included, aged between 0.02 years and 12 years (median age: males 1.25 years; females 1.00 year). A Wilcoxon rank sum test demonstrated no statistically significant difference in age distribution between the sexes (*p* = 0.922; Table 1).

**TABLE 1:** Patient demographics and hydronephrosis by gender and laterality.

Gender	Male (*n* = 44)						Female (*n* = 21)						*p*-value	Overall (*n* = 65)					
	Median	Q1–Q3	*n*	Min–Max	*n*	%	Median	Q1–Q3	*n*	Min–Max	*n*	%		Median	Q1–Q3	*n*	Min–Max	*n*	%
**Age (years)**	1.25	0.0800–6.25	44	0.0200–12.0	-	-	1.00	0.0800–8.00	21	0.0200–12.0	-	-	0.922†	1.00	0.0800–7.00	65	0.0200–12.0	-	-
**Hydronephrosis**	-	-	-	-	-	-	-	-	-	-	-	-	0.273‡	-	-	-	-	-	-
Unilateral	-	-	-	-	23	52.3	-	-	-	-	14	66.7	-	-	-	-	-	37	56.9
Bilateral	-	-	-	-	21	47.7	-	-	-	-	7	33.3	-	-	-	-	-	28	43.1
**VCUG**	-	-	-	-	-	-	-	-	-	-	-	-	0.386‡	-	-	-	-	-	-
Normal	-	-	-	-	16	36.4	-	-	-	-	10	47.6	-	-	-	-	-	26	40.0
Abnormal	-	-	-	-	28	63.6	-	-	-	-	11	52.4	-	-	-	-	-	39	60.0
**Grade unilateral**	-	-	-	-	-	-	-	-	-	-	-	-	0.930§	-	-	-	-	-	-
Rad I	-	-	-	-	3	13.0	-	-	-	-	1	7.1	-	-	-	-	-	4	10.8
Rad II	-	-	-	-	3	13.0	-	-	-	-	3	21.4	-	-	-	-	-	6	16.2
Rad III	-	-	-	-	14	60.9	-	-	-	-	9	64.3	-	-	-	-	-	23	62.2
Rad IV	-	-	-	-	2	8.7	-	-	-	-	1	7.1	-	-	-	-	-	3	8.1
Rad V	-	-	-	-	1	4.3	-	-	-	-	0	0.0	-	-	-	-	-	1	2.7
**Grade bilateral right**	-	-	-	-	-	-	-	-	-	-	-	-	0.205§	-	-	-	-	-	-
Rad I	-	-	-	-	0	0.0	-	-	-	-	1	14.3	-	-	-	-	-	1	3.6
Rad II	-	-	-	-	5	23.8	-	-	-	-	0	0.0	-	-	-	-	-	5	17.9
Rad III	-	-	-	-	11	52.4	-	-	-	-	5	71.4	-	-	-	-	-	16	57.1
Rad IV	-	-	-	-	5	23.8	-	-	-	-	1	14.3	-	-	-	-	-	6	21.4
**Grade bilateral left**	-	-	-	-	-	-	-	-	-	-	-	-	0.725§	-	-	-	-	-	-
Rad II	-	-	-	-	1	4.8	-	-	-	-	0	0.0	-	-	-	-	-	1	3.6
Rad III	-	-	-	-	14	66.7	-	-	-	-	6	85.7	-	-	-	-	-	20	71.4
Rad IV	-	-	-	-	6	28.6	-	-	-	-	1	14.3	-	-	-	-	-	7	25.0

† , Ranksum;

‡ , Chi square;

§ , Fisher’s.

VCUG, voiding cystourethrogram.

Of the 65 patients, 44 (67.7%) were male, and 21 (32.3%) were female, giving a male-to-female ratio of approximately 2:1. This confirmed a male predominance of hydronephrosis in our study population, consistent with previously reported trends in paediatric hydronephrosis.^2,13^ Bilateral hydronephrosis was more common in males (47.7%), while unilateral hydronephrosis predominated in females (66.7%) (Table 1).

The Radiology Grading System was retrospectively applied to all ultrasound examinations to standardise hydronephrosis grading. The Onen Grading system was incorporated for Rad IV/V as Onen had demonstrated correspondence between the two grading criteria.^8^

This dual-grading approach was implemented in this study to rule out inter- and intra-observer variability by providing objective measurements for each grade, defined as follows:

- Rad I: measurement of AP pelvis (AP) 5 mm–7 mm.
- Rad II: AP > 7 mm–10 mm.
- Rad III: AP > 10 mm.
- Rad IV: Parenchymal thickness 3 mm–7 mm.
- Rad V: Parenchymal thickness < 3 mm.

Rad I grading of hydronephrosis was uncommon (10.8% unilateral; 3.6% bilateral on the right only). Among 37 patients with unilateral hydronephrosis, 62.2% (*n* = 23; *p* < 0.001) of patients were graded Rad III (Figure 2). Of the 28 patients with bilateral hydronephrosis, 57.1% (*n* = 16) on the right and 71% (*n* = 20) on the left were graded Rad III (Table 1).

**FIGURE 2:**
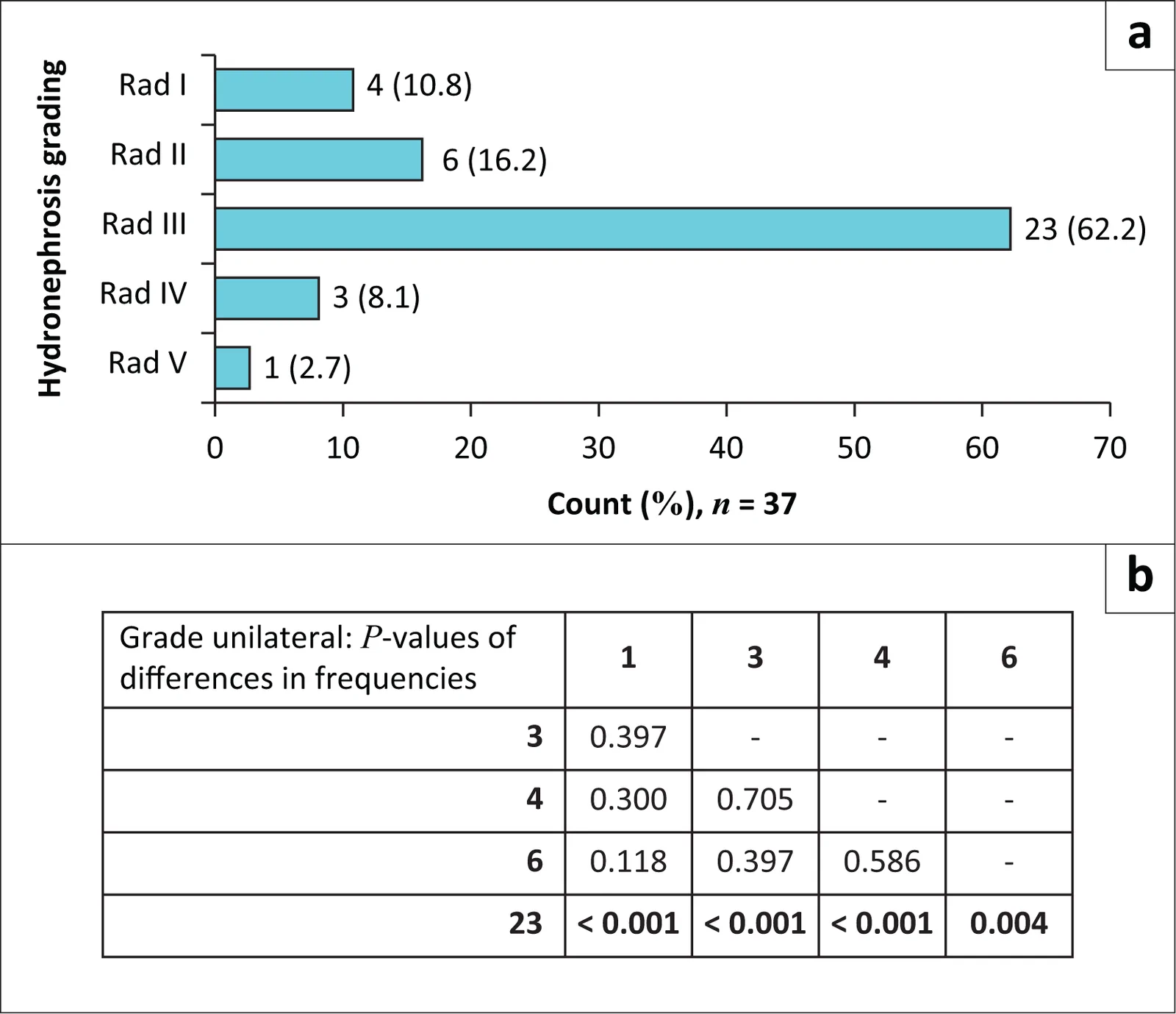
Distribution of unilateral hydronephrosis and pairwise comparison of grade frequencies (a) a bar graph demonstrating frequency distribution and (b) a table representing pairwise comparisons of grade frequencies with *p*-values demonstrating statistical significance.

Figure 3 demonstrates that most patients with bilateral hydronephrosis presented with Rad III (*p* < 0.001) and Rad IV (*p* = 0.002). In cases of bilateral involvement, each kidney was assessed and graded according to its respective severity of hydronephrosis. Individual kidneys were recorded according to their respective grading and lateralisation, accounting for the discrepancy in right-sided and left-sided hydronephrosis in participants with bilateral hydronephrosis.

**FIGURE 3:**
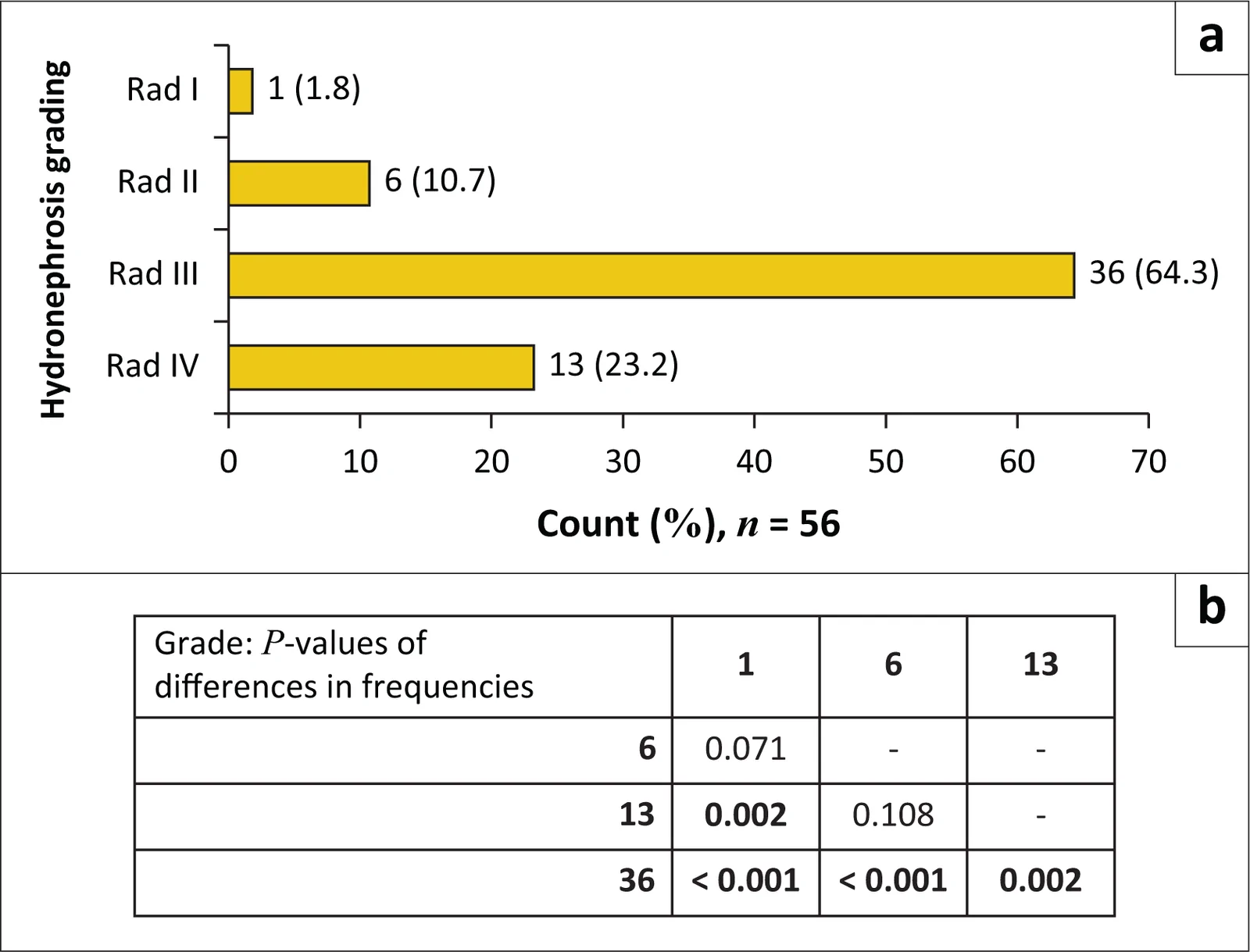
Distribution of bilateral hydronephrosis and pairwise comparison of grade frequencies (a) a bar graph demonstrating frequency distribution and (b) a table representing pairwise comparisons of grade frequencies with *p*-values demonstrating statistical significance.

Rad IV hydronephrosis was observed in 8.1 % (*n* = 3) of unilateral cases and in 23.2% (*n* = 6 on the right and *n* = 7 on the left) of bilateral cases. Only one unilateral case demonstrated Rad V hydronephrosis in a male patient. No Rad V cases were identified among patients with bilateral hydronephrosis.

An underlying aetiology of hydronephrosis was identified in the majority of cases (*n* = 37). Figure 4 further depicts the causes of unilateral and bilateral hydronephrosis, respectively. A discrepancy between the sum of causes and patient numbers was expected, as some patients exhibited more than one abnormality on VCUG. For example, VCUG findings for three cases demonstrated PUV and VUR, one of which also had a bladder diverticulum. Two of these three cases demonstrated abnormal MAG-3 findings: both kidneys demonstrated poor function and slow drainage in the left and adequate function with slow drainage on the right, respectively.

**FIGURE 4:**
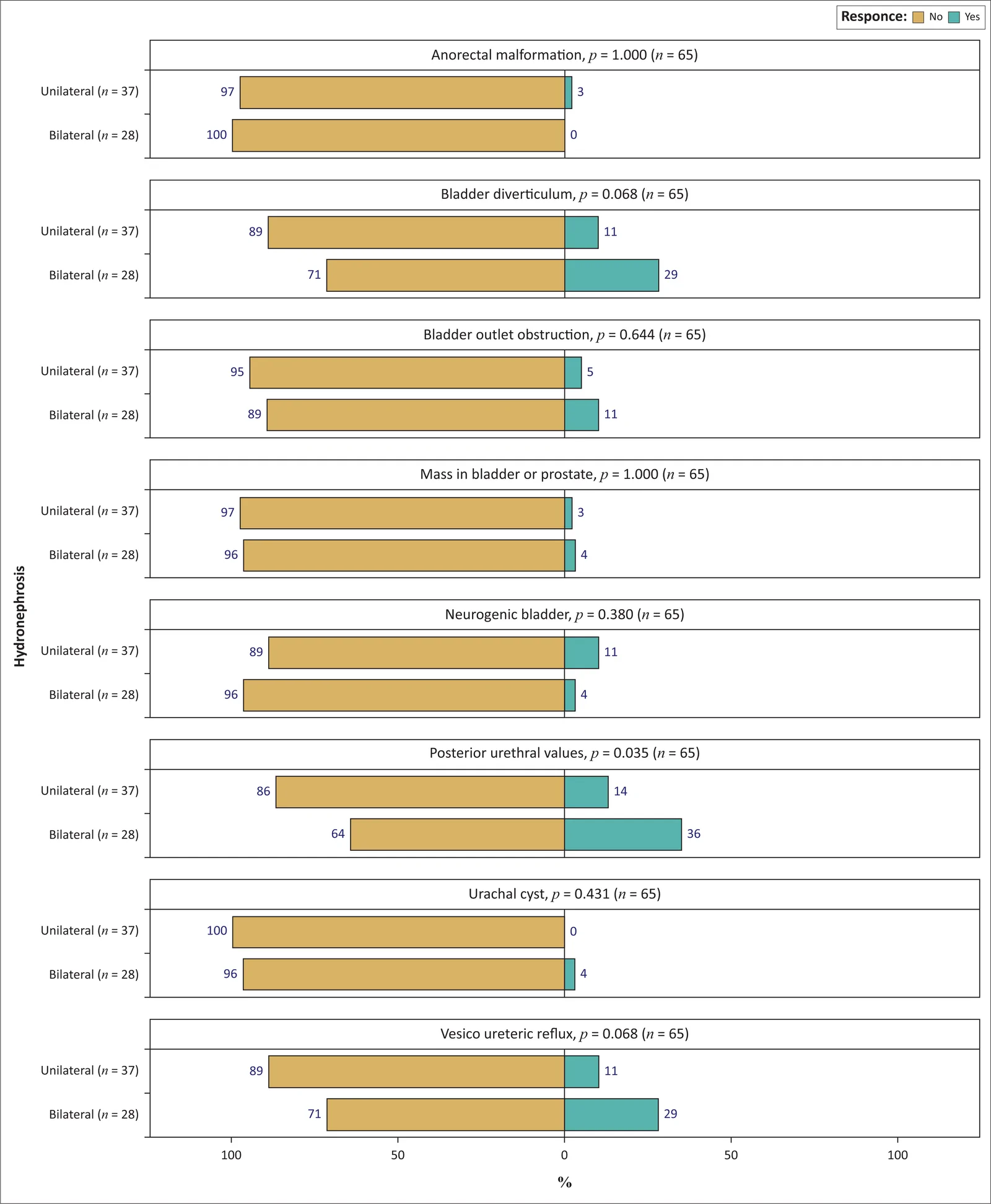
Graphical representation of the causes of hydronephrosis in unilateral and bilateral hydronephrosis.

There were statistically significant differences in the identification of an underlying cause for bilateral compared to unilateral hydronephrosis (*p* < 0.001). Specifically, patients with bilateral hydronephrosis were sevenfold more likely to have an underlying cause reported, when compared to those with unilateral hydronephrosis (odds ratio 7.1; confidence interval: 2.2–28).

The vast majority of pathology corresponded to a Rad III (moderate) hydronephrosis. In our population, aetiology-specific analysis showed that PUV demonstrated that there was a statistically significant significance in patients with bilateral hydronephrosis (36%) compared to unilateral hydronephrosis (14%; *p* = 0.035) (Figure 4; Figure 5). Most of the patients in this subgroup presented with Rad III and Rad IV, with a few Rad II in both unilateral and bilateral hydronephrosis.

**FIGURE 5:**
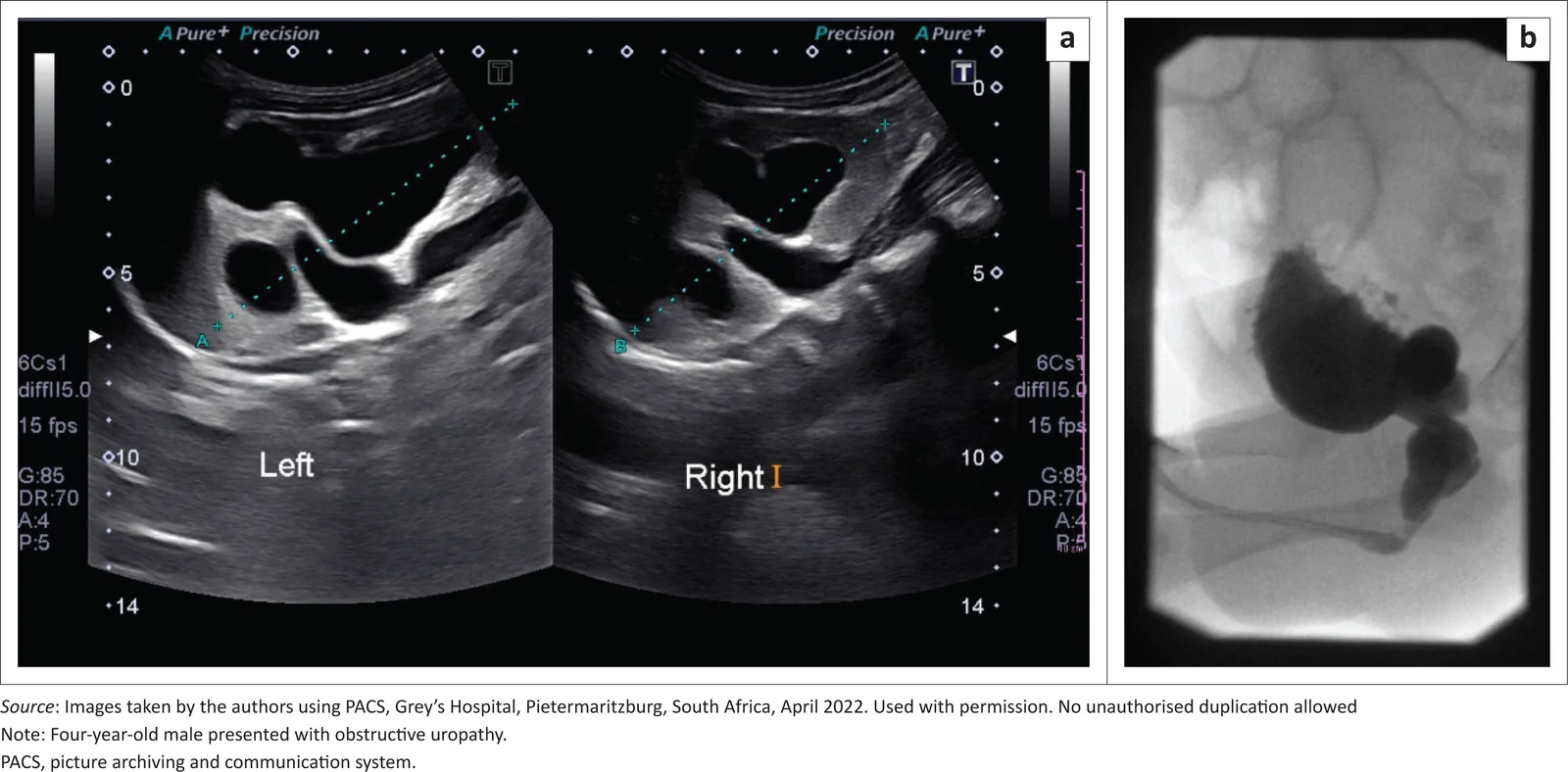
(a) sonographic evidence of bilateral hydronephrosis and (b) voiding cystourethrogram confirmed posterior urethral valves and bladder diverticulum.

Vesicoureteric reflux and bladder diverticulum were more frequently observed in cases of bilateral hydronephrosis (29% each) as opposed to unilateral cases (11% each), though these differences did not meet statistical significance (*p* = 0.06).

Other causes, including bladder outlet obstruction, a pelvic mass, neurogenic bladder (with representative imaging shown in Figure 6), anorectal malformation and urachal cyst, showed no significant difference between unilateral and bilateral groups.

**FIGURE 6:**
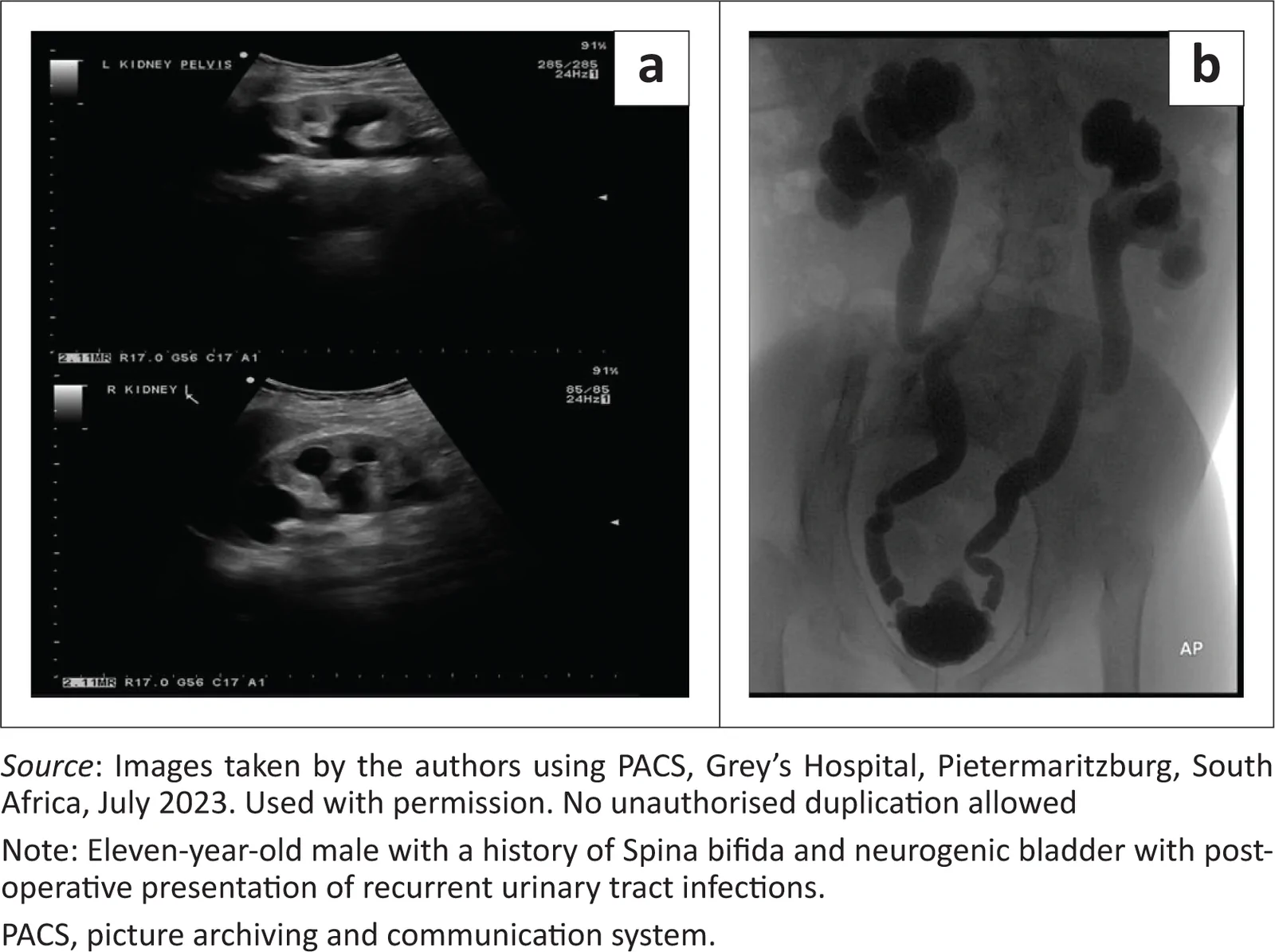
(a) sonographic evidence of bilateral hydronephrosis and (b) voiding cystourethrogram image confirmed vesicoureteric reflux Grade V.

Multiple concurrent causes (≥ 2) were seen more frequently in patients with bilateral hydronephrosis. Conversely, the number of underlying causes per patient differed notably between the groups: 56.8% of unilateral cases had no identified cause, compared with only 14.3% of bilateral cases.

Among the unilateral hydronephrosis group, 47.1% of (*n* = 8) patients demonstrated complete resolution of hydronephrosis on follow-up imaging. Seven of these patients presented with unilateral disease graded Rad I (*n* = 2), Rad II (*n* = 2) or Rad III (*n* = 3) and one patient with bilateral Rad III hydronephrosis. A small subset of patients with congenital abnormalities presented with VCUG evidence of VUR Grade 1 and neurogenic bladder, while others had normal VCUG results and were monitored with sonographic imaging until resolution was confirmed.

Five patients (7%), only one of whom was male, between the ages of 0.3–12 years, underwent VCUG for recurrent urinary tract infections. VCUG findings included VUR Grade V (*n* = 2), chronic bladder outlet obstruction (*n* = 2) and one normal study in the male patient. One documented MAG-3 study for the latter revealed good function and drainage in both kidneys.

Of the 65 patients diagnosed with hydronephrosis, 22 (33%) had available MAG-3 diuretic studies. These patients required functional assessment based on their VCUG evidence of obstruction and clinically significant disease. Correlative findings with VCUG results revealed abnormal lower tract findings in 13 (59%) of these patients, including PUV, VUR or bladder outlet obstruction. The remaining nine (41%) had normal VCUG studies. MAG-3 studies in the former subgroup (*n* = 13) showed delayed excretion or reduced cortical function, while those in the latter indicated non-obstructive hydronephrosis or intrinsic parenchymal disease.

Post-surgical scans demonstrated approximately 50% split renal function per kidney and satisfactory drainage. Fourteen (63%) patients presented with unilateral hydronephrosis secondary to renal dysplasia or non-functioning kidneys with preserved contralateral renal function (split function range: 0–8%). Only one patient had bilateral normal renal function.

Two patients did not attend follow-up hospital visits: one patient had unilateral hydronephrosis with normal VCUG results, and one presented with bilateral hydronephrosis Grade V VUR bilaterally with a neurogenic bladder.

## Discussion

This study retrospectively analysed the underlying causes of hydronephrosis, its distribution and severity based on the Radiology Grading System. Hydronephrosis was predominantly found in males (67.7%), consistent with previous studies.^14,15^

Bilateral hydronephrosis occurred more commonly in males (47.7%)^14,^ while unilateral hydronephrosis predominated in females (66.7%). There is no correlative literature demonstrating unilateral hydronephrosis being common in females, and this finding may warrant further investigation.

The majority of unilateral hydronephrosis cases demonstrated moderate to severe hydronephrosis (Rad III-V), with predominance of Rad III grading (62.2%). In our cohort, patients with bilateral hydronephrosis demonstrated higher grades of hydronephrosis, supporting previous studies noting that the higher grades of hydronephrosis are associated with an increased risk of renal impairment.^16^

Fundamental causes of hydronephrosis identified were PUV, VUR, bladder diverticulum and neurogenic bladder. Among these, PUV exhibited statistical significance in bilateral hydronephrosis.

### Posterior urethral valves

Posterior urethral valves accounted for 36% of patients with bilateral hydronephrosis and 14% with unilateral hydronephrosis (*p* = 0.035). This aligns with published data identifying PUV as one of the leading causes of renal impairment in boys.^17^

It is important to note that 3 VCUG studies displayed PUV and VUR, two of which had documented chronic kidney disease, reflecting the severity of this combination. Coexistence of PUV and VUR increases the patients’ risk of urinary tract infections, reflux nephropathy and subsequently, progressive renal impairment. The importance of early diagnosis and intervention was further reiterated by Peterson et al., who confirmed that 1/3rd of patients may still develop chronic kidney disease within 12 months post-surgical correction.^10,18^

In addition to VUR, bladder diverticula were observed in four patients with PUV. These diverticula are often referred to as renal protective ‘pressure pop-off’ mechanisms protecting the upper tracts by reducing intravesical pressure.^10^ A few patients who underwent follow-up VCUG 3 months post ablation therapy, as a routine procedure,^3^ demonstrated normal findings, indicating decompression. However, persistent VCUG abnormalities following ablation have been reported in other studies, often due to residual valve remnants that may require repeat intervention.^17^

### Vesicoureteric reflux

Although VUR was the second most frequent cause of hydronephrosis in this study, its association was not statistically significant (*p* = 0.068). Vesicoureteric reflux is commonly found in males (64.2%) and presented with bilateral hydronephrosis (29%).^11,12,14^ In our cohort, VUR was associated with Prune Belly syndrome (21.4%), PUV (28.5%) and recurrent urinary tract infections in Spina bifida associated with neurogenic bladder (28.5%).^19^

No documented MAG-3 studies were noted for these patients. It is important to note that Grey’s Hospital does not provide an on-site nuclear medicine service. Mercaptoacetyltriglycine-3 studies are available only through referral to a quaternary institution in Durban, where service demand is high, and access is limited.

From the limited history documented on forms and the small number of files available, it was noted that most patients were followed up with ultrasound as it is more readily available on-site and suitable for serial monitoring. However, a small subset of patients showed interval resolution of hydronephrosis and were subsequently discharged to their local clinic. This emphasises the importance of follow-up every 4–6 months, particularly in mild-to-moderate hydronephrosis.^10^

### Recurrent urinary tract infections

High-grade VUR is a recognised risk factor for UTIs, necessitating further evaluation for potential renal injury.^11^ Hydronephrosis without VUR on VCUG may be secondary to bladder wall thickening potentially impeding drainage.^10^ This represents a diagnostic challenge for both radiologists and clinicians, and it is therefore important to have a low threshold for either re-investigating or further investigating patients who are symptomatic.^10^ Many studies recommend interval sonographic follow-up in such patients.^20^

For asymptomatic patients, Anderson et al. recommend renal ultrasound at 18–24 months, as most significant renal tract abnormalities become evident in this period.^20^ The SASPI guidelines advise that patients with recurrent UTIs undergo MAG-3 or Dimercaptosuccinic acid (DMSA) scans within 4–6 months. Those younger than 2 months old should have a VCUG, and those older than 2 months should have both VCUG and MAG-3 studies performed.^21^

In our setting, due to limited nuclear medicine access, VCUG studies were prioritised in patients with recurrent UTIs. This shows that even though SASPI guidelines were developed for the South African context, their implementation is still constrained by available resources at individual centres.

International guidelines have shifted to a less aggressive imaging approach in children with UTIs. The National Institute for Health and Clinical Excellence (NICE), established 2007, and the American Academy of Paediatrics (AAP), established 2011, both recommend reducing the use of fluoroscopy and nuclear medicine, particularly in the setting of a first typical or characteristic UTI. National Institute for Health and Clinical Excellence (NICE) guidelines further recommended imaging only in atypical cases. This highlights the broader shift to minimise radiation exposure in paediatric patients. In the absence of a universally approved protocol, centres have developed pragmatic approaches suitable for their institution. At Red Cross War Memorial Children’s Hospital in Cape Town, for example, children with a single UTI and a normal ultrasound are not worked up further. Those with recurrent UTIs and a normal ultrasound underwent a MAG-3 study, while patients with abnormal ultrasound findings are triaged by age: VCUG is advised for children under 2 years, whereas children older than 2 years underwent a MAG-3 if there is pelvicalyceal or ureteric dilatation, isolated renal pelvic dilatation or renal size discrepancy. Structural defects or calculi prompt referral to urology, and Doppler abnormalities prompt referral to paediatrics. Importantly, MAG-3 is preferred over DMSA studies due to its lower radiation exposure, and this study recommends radionuclide cystography over VCUG in female patients or males where PUV is not suspected. This aligns with the global trend towards reducing high-dose imaging.^22^

Finally, poor adherence to follow-up attendance is not uncommon in resource-limited settings.^10^ Socioeconomic constraints, transport barriers and overburdened health systems further complicated long-term management and prognosis in this patient population.

### Limitations

This study was conducted at a single tertiary referral centre, and the small sample size may limit the generalisability of the findings. The small sample size may also have reduced the statistical power to detect clinically meaningful associations or differences. Sonographic image interpretation is inherently operator-dependent, which may introduce subjectivity and limit the reliability of retrospective analyses. Non-digitised record-keeping restricted access to patient files, follow- up data and off-site nuclear medicine (MAG-3) results, reducing the ability to examine longitudinal outcomes. In addition, as this study was conducted at a tertiary referral centre, there is a potential for referral bias. Patients referred to tertiary care may have greater disease severity or complexity and may therefore not be representative of the broader disease spectrum encountered in primary- or district-level healthcare settings. Further limitations are noted in the retrospective nature of the imaging data, where unclear images were acquired or no images were stored due to lack of PACS integration with the bedside unit (BSU) ultrasound machine. Reliance on original radiology reports may introduce bias.

Finally, the study period overlapped with the coronavirus disease (COVID) pandemic, during which non-urgent hospital visits and imaging were restricted. This may have affected the small cohort and patient follow-up. These factors further highlight the importance of future prospective, standardised imaging studies.

## Conclusion

This study confirmed that children with bilateral hydronephrosis are more likely to present with usually higher grades of severity of hydronephrosis and identifiable underlying causes, most commonly PUV in boys. Early detection and prompt intervention are imperative to prevent renal disease progression.

Resource constraints pose challenges in fully implementing the SASPI guidelines. Diagnostic pathways should therefore be adapted to institutional capacity while ensuring that the principles of early diagnosis, adequate follow-up and multidisciplinary management are prioritised.
